# Taurodeoxycholate Aggregation Explored by Molecular Dynamics: Primary-To-Secondary Micelle Transition and Formation of Mixed Micelles with Fatty Acids

**DOI:** 10.3390/molecules29245897

**Published:** 2024-12-13

**Authors:** Fatmegyul Mustan, Anela Ivanova, Slavka Tcholakova

**Affiliations:** 1 Department of Chemical and Pharmaceutical Engineering, Faculty of Chemistry and Pharmacy, University of Sofia, 1164 Sofia, Bulgaria; 2 Department of Physical Chemistry, Faculty of Chemistry and Pharmacy, University of Sofia, 1 James Bourchier Ave., 1164 Sofia, Bulgaria; aivanova@chem.uni-sofia.bg

**Keywords:** taurodeoxycholate, myristate, oleate, stearate, self-assembly, micelle structure, all-atom molecular dynamics

## Abstract

Micelles formed by bile salts in aqueous solution are important for the solubilization of hydrophobic molecules in the gastrointestinal tract. The molecular level information about the mechanism and driving forces for primary-to-secondary micelle transition is still missing. In the current study, the micelle formation of 50 mM solutions of taurodeoxycholate (TDC) is studied by atomistic molecular dynamics simulations. It is shown that primary micelles with an aggregation number of 8–10 emerge and persist within the first 50 ns. Then, they coalesce to form secondary micelles with an aggregation number of 19 molecules. This transition is governed by hydrophobic interactions, which significantly decrease the solvent-accessible surface area per molecule in the secondary micelles. The addition of monomers of the sodium salt of fatty acids (FAs), as agents aiding hydrophobic drug delivery, to secondary TDC micelles results in the co-existence of mixed FA-TDC and pure FA micelles. The studied saturated FAs, with chain lengths of C14:0 and C18:0, are incorporated into the micelle core, whereas TDC molecules position themselves around the FAs, forming a shell on the micelle surface. In contrast, the tails of the C18:1 unsaturated fatty acid mix homogeneously with TDC molecules throughout the entire micelle volume. The latter creates a very suitable medium for hosting hydrophobic molecules in the micelles containing unsaturated fatty acids.

## 1. Introduction

Bile salts (BSs) are bio-surfactants synthesized in the human body with important physiological functions in the gastrointestinal (GI) tract [1,2,3,4]. They play a crucial role in solubilization of the lipolysis products (typically monoacylglycerides (MG) and free fatty acids (FA)) by forming mixed micelles with them and thus enhance the digestion process [5]. On the other hand, these species are typically used as components of lipid-based formulations (LBF) for drug delivery due to improved drug solubilization capacity in GI fluids [6]. The absorption of highly lipophilic drugs is boosted when utilizing mixed micellar systems consisting of BSs and polar lipids [4,7]. The solubilization capacity of the mixed micelles varies depending on their exact composition [7,8]. Hence, understanding these structures in more detail is crucial for efficiently designing LBF.

Experimentally, it is found that the unsaturated polar lipids are more efficient to enhance drug solubilization than saturated lipids. For saturated lipids, the degree of solubilization passes through a maximum upon increasing the saturated chain length. The latter is related to the very high solubility in the aqueous phase of the excipients with short chains (<C10) and very low solubility of excipients with the long chains (>C16). Both effects do not allow for the formation of mixed micelles with bile salts, and as a consequence, the solubility of hydrophobic molecules in mixed bile salt–fatty acid micelles passes through a maximum at a chain length of C14 [7]. For unsaturated lipids, the increase in the number of double bonds leads to higher solubilization because of the flexibility of the unsaturated tails [7]. However, the molecular-level structuring of the BS/FA mixed micelles is still not well elucidated.

Molecular dynamics (MD) simulations are a powerful tool for the investigation of experimentally inaccessible phenomena at the molecular level. This method is widely used in the investigations of the GI fluids and drug absorption [9]. In our previous study [10], the kinetics of the formation of pure primary micelles of six different bile salts at physiological conditions (10 mM BS = 8 molecules, 120 mM NaCl, 37 °C) was studied by atomistic MD simulations. We showed that the aggregation was a hierarchical process and that its kinetics depended both on the number of hydroxyl groups (two or three) attached to the steroid skeleton and on the type of BS conjugation (glycine or taurine). One of the conclusions was that the formed micelles were stabilized mainly by hydrophobic interactions. Intermolecular hydrogen bonds served to speed up the aggregation of conjugated bile salts but not to stabilize the primary micelles. This was the first computational work at such a low concentration of the surfactants, corresponding to that at physiological conditions. Other studies also stated that the main intermolecular interactions within the primary micelles were the hydrophobic ones and outlined hydrogen bonds as the main factor shaping the secondary micelles by influencing their size, structure, and dynamics [11,12,13]. Along the same lines, Turner et al. concluded that the aggregation of glycocholate was driven by hydrophobic interactions between the non-polar phase of the bile salt steroid skeleton, obtaining micelles with an average aggregation number, *N*_agg_ = 8.5 molecules [14]. These conclusions were based on atomistic simulations with a united atoms force field conducted for 36 ns at 37 °C. The authors discussed that the continuous association of monomers to the formed micelles was a result of the hydrogen bonds between the three OH groups in the skeleton of the glycocholate. In a coarse-grained simulation study, the association of monomers into existing micelles was listed as the main mechanism of primary micelles growing to secondary structures [15]. Recently, Pabois et al. studied by MD simulations the morphology of assemblies of two bile salts–sodium taurocholate and taurodeoxycholate [16]. They applied atomistic simulations with the force field CHARMM36 on model systems containing 10, 20, and 50 mM BS (12 or 25 molecules) at physiological conditions for 150 ns. They observed the formation of ellipsoidal micelles by TDC with *N*_agg_ = 11 ± 2 and radius 1.2 nm stabilized by hydrophobic interactions and an increase in the size of the micelles upon increasing the concentration. To assess the intermolecular interactions within the micelles, the authors analyzed the contacts between different atoms from the hydrophilic and hydrophobic moieties of the molecules and obtained much more contacts between the hydrophobic phases than between the hydrophilic parts. However, the mechanism of the formation of these micelles was not discussed and remains unclear.

The next step in the understanding of the behavior of the biorelevant aggregates is the detailed study of the mixed bile salt/lipolysis product micelles. Turner et al. used united-atom MD simulations to study the aggregation behavior of bile salt/oleic acid mixed micelles [14]. Their mixed model system contained 22 glycocholate molecules (~29 mM) preassembled into an aggregate, 9 monomers of sodium oleate, and 150 mM NaCl, which were simulated in water for 36 ns at 37 °C. The authors found that the micelle increased in size when oleate was incorporated into it, creating a fluid-nature mixed micelle. The oleate anion penetrated between the outer layers of bile salt, making its way into the central core of the micelle, which was enveloped by a hydrophobic network created by the steroid backbone of the bile salts. Most of the other simulations on this topic are performed with coarse-grained MD. Using this method with the force field Martini at physiological conditions, Parrow et al. studied mixed micelles of bile salts (glycocholate, glycodeoxycholate, taurocholate, and taurodeoxycholate), phospholipids, free fatty acids, and cholesterol in different ratios [17]. The latter corresponded to experimental data about the composition of aspirated duodenal fasted-state samples in fasted and fed states of 20 healthy volunteers. They observed the formation of ellipsoidal micelles with sizes ranging from 4 to 7 nm for 3 μs of simulation time. By the same approach, Tuncer et al. investigated the evolution of mixed micelle formation under physiological conditions in duodenum both at fasted and fed states of the GI fluids [18]. They used cholate and POPC as model bile/lipid described with the force field Martini. The authors observed the formation of slightly ellipsoidal micelles independent of their size, which varied between 1.3 nm and 1.9 nm. Applying a radial density distribution analysis, it was suggested that the core of the micelles was composed of POPC tails, the glycerol residues were laid on the micelle surface, and the charged phosphate and choline groups were orientated toward the surrounding medium, where they were completely solvated with water [18]. In a subsequent investigation of the same authors, the effect of the chain length and saturation of the fatty acids on the properties of the mixed micelles was studied [19]. Their model systems contained 193 cholic acid, 48 POPC, and 193 FA (laurate, stearate, oleate, and linoleate) molecules in ratio 20:5:20 and 150 mM NaCl, which were simulated in water for 8 μs at 37 °C. They established the formation of mixed micelles coexisting with pure/small BS micelles and BS monomers. Mixed micelles with *N*_agg_~100, *r*_g_~2.2 nm and slightly elliptic spheroidal structures were obtained with C12:0, C18:0, and C18:1, whereas *N*_agg_ = 248 and *r*_g_ = 3.85 nm were registered in the presence of C18:2, which formed two huge worm-like mixed micelles as separate phases. Independent on the length and presence of a double bond in the hydrocarbon chain of the FAs, the authors obtained that the core of the micelles was occupied by POPC and FA tails and the surface was covered by cholate and the charged head groups of the lipids. They discussed also that the core density of the micelles decreases with an increase in the unsaturation degree of the chains because of their enhanced flexibility.

It is well known that the main interactions between BSs are hydrophobic attractions driving the formation of primary micelles, and hydrogen bonds assist their structuring into secondary micelles, which have not yet been unanimously established [1]. However, almost nothing is discussed in detail about the mechanism of the formation of the pure BS micelles. With respect to the mixed aggregates, it is suggested that the main forces acting between different kinds of molecules are hydrophobic interactions. Overall, in most of the works, the focus is on the composition and morphology of the micelles or on the solubilization of drug molecules therein. To shed light on the reasons for higher solubilization capacity of the mixed micelles with unsaturated chain moieties, the intermolecular interactions inside the mixed micelles should be studied in more detail. Clarifying the molecular origin of the experimentally observed solubilization phenomena with respect to the effects of the chemical structures of the used excipients will allow for a much better understanding of the systems and facilitate the creation of efficient LBF.

The major goals of the current study are (1) to determine the kinetics of formation of secondary micelles in BS solutions and clarify the underlying mechanism driving this process and (2) to determine the structure of the mixed micelles formed from bile salts and fatty acids with different chain lengths (C14:0 and C18:0) and degrees of unsaturation (C18:0 and C18:1) in order to elucidate the reason for their different capacities to solubilize the hydrophobic drugs in the hydrophobic interior [7]. To obtain insight into these issues, atomistic MD simulations address the structure of the micelles formed by TDC and FAs and the intermolecular interactions therein.

The paper is organized as follows: the studied molecules, simulated model systems, and used computational protocol are described in the next section. Then, the obtained results for pure bile salt micelles are summarized and discussed in the first subsection and the mixed micelles with fatty acids in the second one. First the morphology and then the intermolecular interactions are presented for both types of micelles. The main conclusions are outlined at the end.

## 2. Results and Discussion

### 2.1. Pure TDC Micelles

#### 2.1.1. Kinetics and Mechanism of Formation

**Figure 1:**
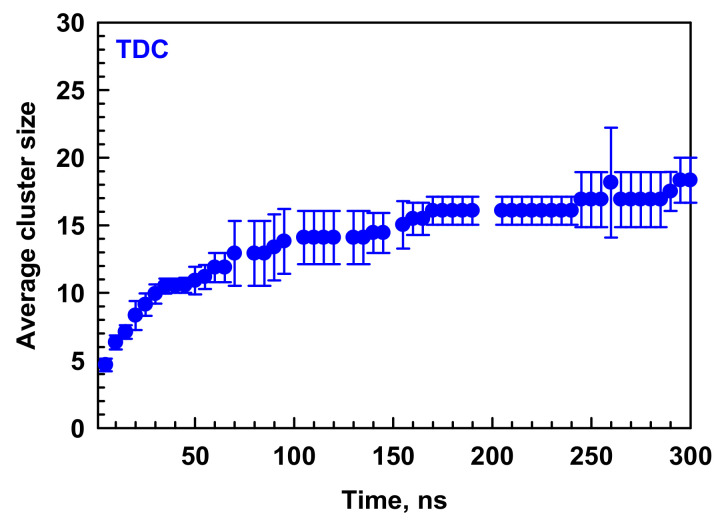
The average cluster size of TDC as a function of simulation time. Average values and standard deviations of the results obtained from two independent simulations are calculated.

The self-assembly of the bile salts into micelles is a spontaneous process in an aqueous solution, which is driven by the dispersive attraction between the molecules to screen their hydrophobic moieties from the surrounding water. In our previous work, we monitored the micellization of eight molecules in the simulation box, which yielded a pentamer and a trimer for TDC, known as primary micelles in the literature [20]. The current study is focused on the secondary micelles. To reveal how they are formed, in addition to the last 10 ns of the simulations, we analyzed in detail the micelles obtained in the initial stage of the simulations (37–47 ns), where there are still primary micelles (1 trimer, 1 pentamer, 1 hexamer, and 2 decamers) and a few secondary ones with aggregation numbers 14, 16, 17, and 19. The illustrations of the micelles in the periodic box at 37 ns and 300 ns are shown in Figure S1 in the Supplementary Materials. The average *N*_agg_ of TDC at 300 ns is 19 ± 2, which is in very good agreement with the experimental data in the literature, *N*_agg_ = 12–19 [1,20,21,22]. Pabois et al. report *N*_agg_ = 11 ± 2 of TDC micelles from MD simulations at 150 ns [16]. At this time, a very similar value of 14 ± 2 is obtained in our simulations (Figure 1). In accordance with our previous study, the kinetics of micellization is very fast at the beginning of the simulations due to the presence of many monomers, where the average size of the clusters increases steeply in the first 30 ns. Its further increase is related to the coalescence of two primary micelles forming secondary structures (Figure 1).

**Figure 2:**
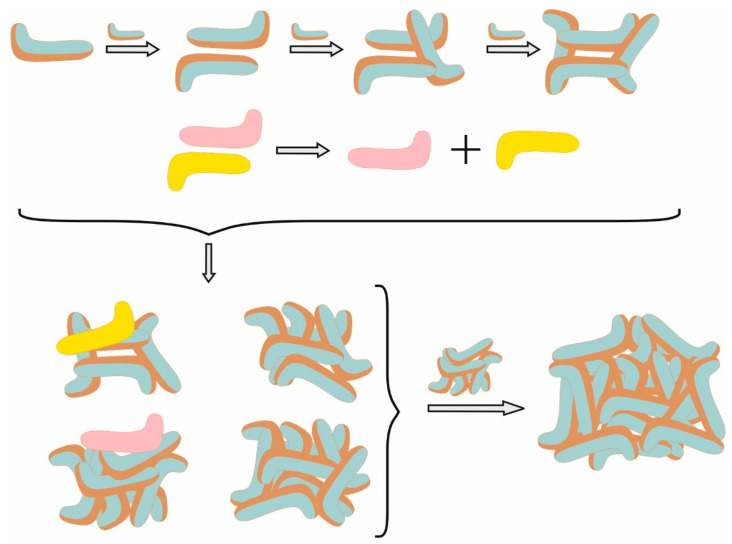
A schematic illustration of the micellization of the TDC formation of primary and secondary micelles. Cyan is the hydrophilic face; brown is the hydrophobic face of the skeleton; and yellow and pink molecules are TDC monomers released from dimers and associated with two different micelles.

This steep increase in the average cluster size is accompanied by a sharp decrease in the number of monomers in the same time frame of the simulation (Figure S2A in the Supplementary Materials). Very quickly, all the monomers are arranged into dimers, trimers, and tetramers, the number of which passes through a maximum as a function of time (Figure S2B in the Supplementary Materials). The subsequent decrease in their number is related to the formation of larger micelles with *N*_agg_ up to 13 by the addition of existing monomers or such from disintegrating dimers and trimers. It is found that the initially formed dimers and trimers are not only stable enough to keep growing, but they also release monomers that incorporate into other larger micelles. However, such a phenomenon is not observed for the micelles containing four or more molecules. They either take monomers to keep growing or associate with larger micelles, forming secondary ones. An illustration of the mechanism of micellization of TDC is shown in Figure 2.

At this stage, the average size of the clusters increases stepwise (Figure 1): there are periods with a constant size, then it abruptly increases, followed by the next stationary period, and so on. The reason for these jumps between two quasi-equilibrium states is the merging of two micelles with different sizes to form a secondary one. This aggregation stage is much slower than the initial one because of two factors controlling the kinetics of micellization: slower diffusion of the clusters and larger distances between them (analogous to the speed of motion and distance for passing, which determine the characteristic time of the macroscopic process). These two factors are very favorable in the presence of many monomers, which occupy almost the entire simulation box and thus maximize the probability of encountering each other. Moreover, it is much easier for two or three monomers to match because each part of the molecules is accessible for interactions, allowing for the instantaneous formation of clusters. However, the primary micelles are larger than monomers, and they diffuse slower than them. In addition, in order to assemble into secondary structures, the molecules within the micelle need to rearrange to create an appropriate interaction area. Consequently, the contact of two primary micelles does not lead immediately to the formation of secondary micelles. That is why the kinetics of the formation of secondary micelles is slower (10^2^ ns) than that of the assembly of the primary structures (10^1^ ns). It should be mentioned that there are no free monomers in the simulation box after 30 ns, but they can be released from dimers or trimers and associate with the large micelles as well, which are mainly formed upon the coalescence of two smaller clusters.

#### 2.1.2. Morphology of the Micelles

**Figure 3:**
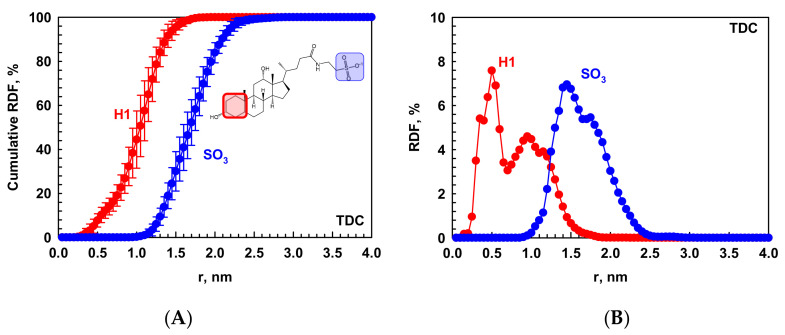
(**A**) The cumulative number RDF in % and (**B**) distributive RDF normalized to the area under the curve with respect to the geometric center of each micelle for the COG of SO_3_ (blue circles) and COG of *H*1 (red circles) in TDC. The calculations are performed for each micelle separately and averaged over the last 10 ns of the simulation and over the different micelles.

The morphology of the micelles is determined quantitatively from the RDF of the two terminal groups of the molecules with respect to the geometrical center (COG) of the micelle. For TDC, these are the first six-membered ring and the sulfonate group, denoted as *H*1 and SO_3_ in Figure 3, respectively.

As expected for TDC micelles, the hydrophobic fragment is closer to the micelle core than SO_3_, the latter hydrophilic part being oriented toward water. Depending on the size of the micelle, the most probable distance of the molecular fragments from the center is different. That is why there are two peaks in the RDF profiles in Figure 3B, which are calculated separately and averaged for all formed micelles. It is important to note that there is a hydroxyl group attached to the mentioned first six-membered ring in the hydrophobic part of the molecule, which takes part in the hydrophilic face of the skeleton. Such not distinctly separated hydrophilic and hydrophobic parts of the bile salts, as opposed to the conventional surfactants, interfere with the formation of much bigger micelles because one side of the skeleton seeks shielding from the water, but the other side, being hydrophilic, is prone to interact with the solvent. That is why these structures are very flexible, with profound dynamics of the molecules, and attaining favorable structuring for both faces of the molecules is very challenging. The presence of other molecules—entirely lipophilic or amphiphilic but with well-defined hydrophobic and hydrophilic parts—is beneficial for the bile salts to solubilize them by creating a hydrophobic phase to interact with. This scenario is discussed in more detail in Section 2.2.

#### 2.1.3. Driving Forces for the Micellization

**Figure 4:**
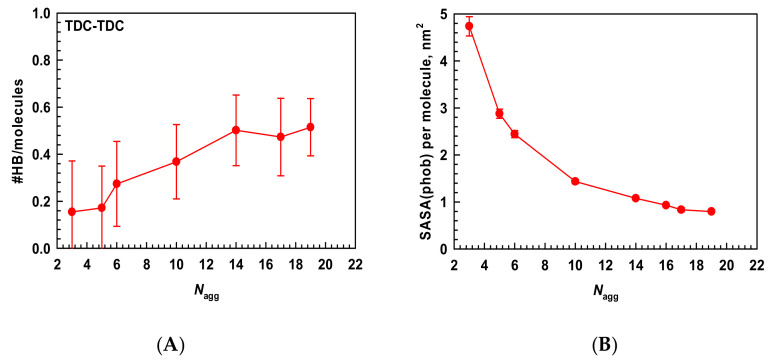
(**A**) The number of intermolecular hydrogen bonds per TDC molecule and (**B**) the SASA of the hydrophobic parts (steroid skeleton and CH_3_ groups) per molecule in the micelles with different aggregation numbers averaged in the period between 37 and 47 ns. The SASA is calculated at a default radius of the solvent probe of 0.14 nm for water molecules.

Are hydrophobic interactions or hydrogen bonds the major forces for the formation and stabilization of the relatively small secondary micelles? How important are they? To answer these questions, we performed a detailed analysis of the micelles formed between 37 and 47 ns, where there are aggregates with aggregation numbers between 3 and 19 in the simulation box. The mechanism of the formation of the smallest clusters has already been addressed in our previous study [10]. Initially, we used the same approach as in the first work to assess the intermolecular interactions in the micelles with different sizes. However, this yielded very similar results independent of the aggregation number. In all of the secondary micelles, the minimum distance between the molecules is 0.225 nm, indicating an entirely hydrophobic core of the micelles, devoid of water. An example of the distribution of the minimum distances between the molecules is given in Figure S3 of the Supplementary Materials. It means that this is the characteristic shortest distance between the molecules arranged into a micelle, irrespective of its size. The next step was to determine the intermolecular hydrogen bonds. The number of hydrogen bonds (normalized to 1 molecule) as a function of the aggregation number is shown in Figure 4A. It is found that there is some increase in the count of HBs with the aggregation number of the micelles, but it is not marked. Moreover, there are no stable HBs for the whole period of 10 ns. This indicates that hydrogen bonds are not the driving force for the formation of the secondary micelles. Next, we analyzed the SASA (normalized to 1 molecule) of the hydrophobic parts of the molecules in the different micelles to assess how well they are shielded from the water (Figure 4B). It is evident that the SASA decreases considerably with the aggregation number. It is 4.7 nm^2^ for the trimer and reaches almost a plateau region after *N*_agg_ = 16 at a value smaller than 1 nm^2^. There is almost 5 times difference between the SASA of the smallest primary and the largest secondary micelles. This reveals the driving force of the second stage in the micellization of the bile salts—hydrophobic interactions. The relatively large hydrophobic area of the molecules exposed to water in the primary micelles forces them to associate more molecules and form the secondary micelles. At a high concentration, this reorganization is much more favorable since it allows for much better screening of the hydrophobic moieties. In conclusion, the hydrophobic interactions are the driving force for the formation and stabilization of both primary and secondary micelles of the bile salts, and the hydrogen bonds are a consequence of the molecules being in contact and containing appropriately placed hydrogen bond donors and acceptors.

### 2.2. Mixed Micelles of TDC and Fatty Acids

**Figure 5:**
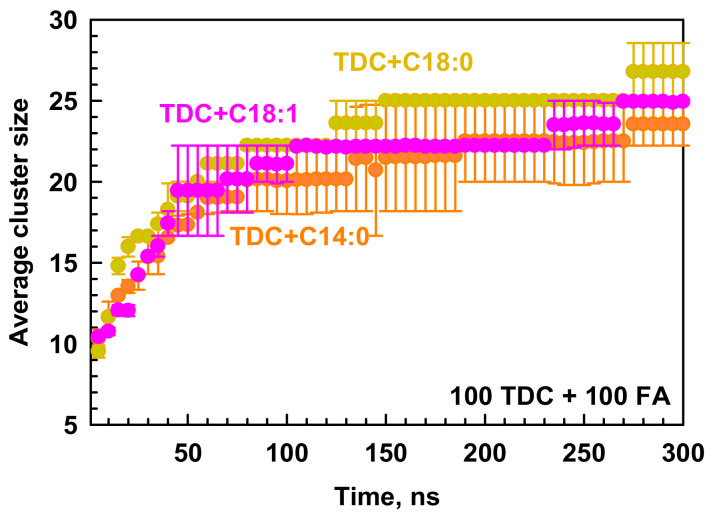
The average size of clusters as a function of the simulation time in the TDC systems with sodium myristate (orange circles), stearate (yellow squares), and oleate (pink rhombi). Average values and standard deviations of the results obtained from two independent simulations are calculated.

When fatty acids are added, the formation of six or more clusters by all studied molecules is witnessed. In this case, either the assembly of mixed micelles of TDC and FAs or the solubilization of FA molecules in TDC micelles is observed, which is also experimentally known [7,16]. Snapshots at 300 ns of the production runs of each model system in the presence of fatty acids are presented in Figure S4 in the Supplementary Materials. In addition, the formation of pure FA aggregates in the aqueous phase can be seen, which means that the available amount of TDC is not enough to solubilize all the present FA molecules. It is visible that the stearate molecules are very well ordered, as in a crystal (Figure S4B). Good ordering is observed for the myristate (Figure S4A), too, but hardly any for the oleate (Figure S4C). The kinetics of aggregation in terms of the average cluster size is presented in Figure 5. Again, the micelles are formed gradually and steeply at the beginning of the simulations for ca. 60 ns, which is related to the quick incorporation of the FA monomers to already exciting TDC micelles or to the assembly of FA molecules into separate pure aggregates. The results obtained at the end of the simulations (at 300 ns) are used to calculate the average aggregation number from different simulations of the same system (Table 1) and the exact composition of each micelle (Table S1 in the Supplementary Materials). The addition of the same number of FAs to TDC micelles increases the aggregation number to 25 independently of the used FAs. In all systems, six or eight mixed micelles with different ratios between TDC and FAs and between one and three pure lipid micelles are formed (Table S1 in the Supplementary Materials). The average fraction of the lipids incorporated into TDC micelles is ca. 40%.

Note, because of the short plateau region in the average cluster size in the last 10 ns of the simulations, we extended the simulations up to 700 ns for only one trajectory of each system (Figure S5 in the Supplementary Materials), which did not lead to qualitatively new outcome. Hence, the results presented below regarding the micelle properties are for the structures in the time range of 290–300 ns where micelles with *N*_agg_ varying between 18 and 50 molecules were analyzed separately.

#### 2.2.1. Micelle Composition and Morphology

Among the most discussed properties of the bile salt micelles are their size and shape. However, there is no unanimous opinion, neither in the experimental nor in the simulation works. The reason for this discrepancy is the intensive dynamics of the molecules inside the micelles and their much smaller sizes compared to micelles of conventional synthetic surfactants. Previously, we found that pure BSs assemble mainly in irregular sphere or ellipsoidal-like morphologies, which is also one of the most discussed phenomena in the literature, obtained from experiment [16] or MD simulations [13,15]. To determine the shapes of the secondary micelles of TDC and the mixed micelles with FAs in the current study, the ratios between moments of inertia are calculated (Table 1). It is known that *I*_1_/*I*_2_ ≈ 1 and *I*_2_/*I*_3_ ≈ 1 correspond to spherical shapes, *I*_1_/*I*_2_ ≈ 1 and *I*_2_/*I*_3_ ≪ 1 to disc-like shapes, and *I*_1_/*I*_2_ ≪ 1 and *I*_2_/*I*_3_ ≈ 1 to rod-like shapes. In the current study, for all micelles, *I*_2_/*I*_3_ ≈ 0.88 is obtained, whereas *I*_1_/*I*_2_ varies in a wide range. It is 0.80 for TDC pure micelles, 0.87 for the micelles containing C18:0, and ≈0.75 for those with C14:0 and C18:1. It means that all micelles have an irregular shape. It is sphere-like for TDC and TDC + C18:0 aggregates and ellipsoid-like for the mixtures with C14:0 and C18:1, which is in good agreement with SANS measurements [16]. The main point here is that the addition of C18:0 does not change the shape of TDC micelles, but C14:0 and C18:1 slightly elongate them. A similar trend with respect to the solubilization capacity of these micelles was determined experimentally; C18:0 does not change the solubilization of hydrophobic drugs, but both C14:0 and C18:1 increase it by forming mixed micelles [7]. More about the solubilization capacity of the formed micelles is discussed below in the text.

**Table 1:** The fraction of the FAs in the mixed micelles, the aggregation number, the average radius of gyration, the volume of the micelles, the ratio between moments of inertia, and the corresponding shape of the micelles.

System	TDC	TDC + C14:0	TDC + C18:0	TDC + C18:1
FA in the mixed micelle, %	-	42 ± 15	41 ± 14	44 ± 23
*N*_agg_	19 ± 2	25 ± 11	25 ± 8	25 ± 6
Experiment	18 *; 15.9 **	-	-	-
Radius of gyration, *r*_g_, nm	1.25 ± 0.07	1.35 ± 0.20	1.39 ± 0.17	1.46 ± 0.14
Volume, nm^3^	8.20 ± 1.30	11.00 ± 5.02	11.50 ± 3.21	12.63 ± 5.26
*I_1_*/*I*_2_	0.80 ± 0.06	0.75 ± 0.04	0.87 ± 0.11	0.76 ± 0.04
*I*_2_/*I*_3_	0.88 ± 0.04	0.89 ± 0.04	0.88 ± 0.02	0.85 ± 0.04
Shape	Irregular sphere	Irregular ellipsoid	Irregular sphere	Irregular ellipsoid

* Ref. [20]; ** Ref. [21].

To determine the size of the aggregates, the radius of gyration is calculated. The obtained results are presented in Table 1. Pertaining to the smallest aggregation number of pure TDC micelles, their radius is the smallest, *r*_g_ = 1.25 ± 0.07 nm, which is in excellent agreement with the MD simulations of Pabois et al. at similar conditions (50 mM TDC, 150 mM NaCl, CHARMM, 300 K, 150 ns production run), *r*_g_ = 1.11 ± 0.10 nm [16]. The addition of FAs slightly increases the size of the aggregates, which is related to the increased aggregation number. For identical *N*_agg_, the radii of gyration of the mixed micelles with different lipids slightly vary in the following way: 1.35 ± 0.20 nm, 1.35 ± 0.20 nm, and 1.46 ± 0.14 nm for C14:0, C18:0, and C18:1, respectively. The reason for that is the increased size of the molecules in the same order, which is observed experimentally for the mixed micelles of BS and triglycerides with different chain lengths [8]. The same effect of chain length is also registered in the work of Pabois et al., where C8:0 and C18:0 FAs are compared [16].

**Figure 6:**
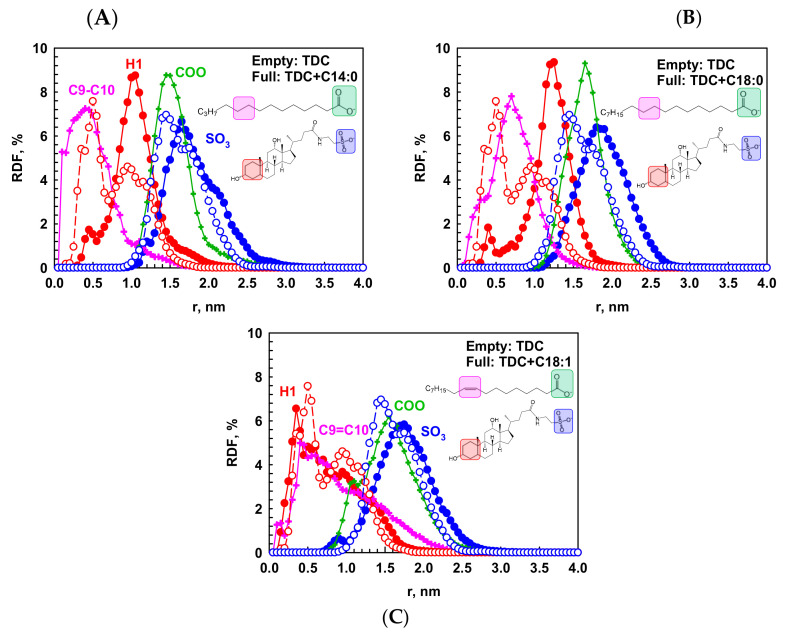
The RDF normalized to the area under the curve with respect to the geometric center of each micelle for SO_3_, blue circles; for the first 6-membered ring (*H*1), red circles in TDC; for COO, green crosses; and for C9–C10, pink crosses in FAs, in the systems of (**A**) TDC + C14:0, (**B**) TDC + C18:0, and (**C**) TDC + C18:1. Empty points are in the RDF for TDC micelles without fatty acids. The analyzed atomic groups are colored in the molecular structures shown on the plots. The calculations are carried out for each micelle separately and averaged over the last 10 ns of the simulation and over all different micelles.

**Figure 7:**
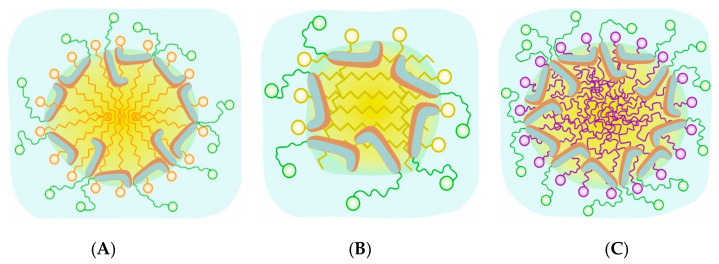
The illustration of the placement of the various molecules in the mixed micelles of TDC (skeleton, cyan and brown, and taurine chain, green) with (**A**) myristate in orange, (**B**) stearate in yellow, and (**C**) oleate in purple.

To quantify the ordering of the molecules inside the micelles, we calculated the RDF (cumulative and distributive) between the geometrical center of the micelles and those of different fragments of the two types of molecules: SO_3_ and first six-membered ring of TDC; C9-C10 and COO groups in FA molecules (Figure 6 and Figure S6 in the Supplementary Materials). The most probable position of the hydrophobic part of TDC is at 0.48 ± 0.12 nm. Interesting reordering of the molecules appears upon addition of different FAs. The chain of C18:0 is completely solubilized into the TDC micelle, shifting *H*1 to 1.06 ± 0.30 nm from the core because of the occupation of its former position by the C18:0 chain. Similar reordering is observed upon the addition of C14:0 as the *H*1 is shifted to 0.94 ± 0.22 nm. However, the picture is different in the case of the unsaturated chain. It is located at the same position as the hydrophobic part of TDC, which is shifted only 0.1 nm to the same location with the double bond. The illustration of the structure of the mixed micelles is presented in Figure 7. This particular behavior of C18:1 is related to the curvature of the chain due to the double bond but not only. The phenomenon is explained in more detail in the next section. 

Based on the characteristic distances of the functional groups (see the Supplementary Materials), it may be concluded that the carboxylate is always ‘underneath’ the sulfonate, irrespective of the FA chain length. However, the sulfonate has a rather fixed preferred position with a maximum between 1.5 and 1.8 nm from the center of the micelle in the pure and in the mixed micelles, respectively.

In summary, the aggregation number of the mixed micelles is larger than that of the pure ones (19 vs. 25), and it does not depend on the used fatty acid. The saturated chains are completely solubilized into the micelles, creating the hydrophobic core, whereas the double bond fragments in C18:1 interact with the hydrophobic part of the bile salt and forms a homogeneous fluid-like mixed micelle. The radius of gyration of the micelles increases in the following way: TDC < TDC + C14:0 < TDC + C18:0 < TDC + C18:1. Regarding the ratios of the moments of inertia, the shape of the pure TDC and mixed with C18:0 micelles resemble an irregular sphere, while the aggregates with C14:0 and C18:1 are more akin to irregular ellipsoids.

#### 2.2.2. Intermolecular Interactions in the Micelles

It is of interest to investigate in more detail the different localization of the C18:1 chain in the micelles compared to the saturated ones. It is clear that there are hydrophobic interactions between the steroid and alkyl residues, but also, hydrogen bonds are formed even with the OH group attached to the first six-membered ring (*H*1). We looked at the partial atomic charges of these residues of all molecules and calculated the total charges of the *H*1, C_2_H_2_, and C_2_H_4_ in TDC, unsaturated FAs, and saturated FAs, respectively (Table 2). Note, here we use the values of the RESP charges, which are derived from the electrostatic potential of the molecules calculated with quantum mechanical method as described in Section 3. It is established that the charge of *H*1 is positive, whereas it is negative for the FA chains, which means that electrostatic attraction is plausible between these residues. According to the Coulomb rule, the electrostatic interaction is proportional to the product of the two attracting charges. To examine the effect of the partial charges of these residues, their product is presented in Table 2. It is seen that it is almost zero for the saturated FAs but it is −0.121 for C18:1, which is 5 times higher than for C14:0 and 38 times higher than for C18:0. This is an indication of an extra interaction (in addition to the hydrophobic attraction) between TDC and C18:1, not so pronounced with the saturated chains.

**Figure 8:**
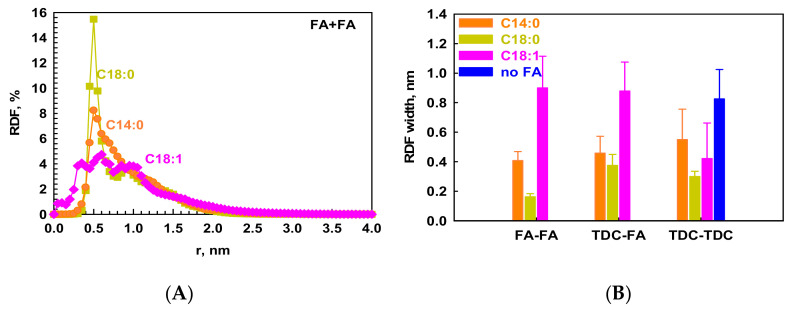
(**A**) The RDF between the center of geometry of pairs of the FAs in the systems of TDC with C14:0 (orange circles), C18:0 (yellow squares), and C18:1 (pink rhombi). (**B**) The full width at half maximum (FWHM) of the RDF profiles between FA-FA, TDC-FA, and TDC-TDC calculated for each micelle separately and averaged.

It seems that this additional interaction between TDC and C18:1, combined with the higher flexibility of the unsaturated chain, makes possible the formation of well-homogenized mixed micelles. So far, the interactions between BSs and lipids have not been discussed in detail in the literature. To assess that, we calculated the RDF profiles between the geometrical centers of the different types of surfactants (on a per-molecule basis) for each micelle separately. The obtained raw data are plotted in Figure S7 in the Supplementary Materials. The average profiles for FA-FA interactions from all formed micelles in each system are presented in Figure 8A and those for TDC-TDC and TDC-FA in Figure S8A,B in the Supplementary Materials, respectively.

Quantitatively, the flexibility of C18:1 is illustrated very well by these RDF profiles (Figure 8A, pink rhombi). It can be seen that there is no well-expressed peak in the RDF between both pairs FA-FA (Figure 8A) and FA-TDC (Figure S8B in the Supplementary Materials) in comparison with the RDF profiles for saturated chains where the peaks are much sharper. The latter are much more ordered with respect to each other, especially C18:0, which is illustrated by the separate snapshots of all micelles in Figure S9 of the Supplementary Materials. The RDF peak is the sharpest and the most intensive for C18:0, followed by C14:0, which also features a well-defined but less intensive peak, indicating weaker interactions between the C14:0 tails compared to C18:0 because of the contribution of each CH_2_ to the interaction energy [23]. Concerning the interactions between FA and TDC, estimated also by the RDFs (Figure S8B in the Supplementary Materials), a well-expressed peak is established, which is the same with the two saturated FAs and spans from 0.35 nm to 1.5 nm distance between the molecules. This well-defined interaction in a comparatively small distance range is related to the tight ordering of the saturated chains. In contrast, the peak for the unsaturated FAs is lower but wider, which signifies a lack of structuring of these flexible molecules spread along the micelles in many conformations. Note that the presented RDF profiles are averaged for all micelles formed in the model systems, but for a clearer illustration of the profiles, the error bars are not shown. The main difference between the RDFs of the separate micelles is their intensity but not the profile and the position of the peaks, as the differences are related to the various ratios between the two types of molecules forming the micelles. From these RDF profiles, the width (*w*) of the peak is calculated (Figure 8B), which indicates how rigid the structure within the molecules that interact is (see the discussion and Figure S10 in the Supplementary Materials). These data confirm the rigidity of the C18:0 tails at the applied conditions. That is why the solubilization capacity of this system is so low. Rigid entities are not able to host new molecules because the process of rearranging the frozen molecules will require significant energy. Note, there are molecules at shorter distances than the other even in the same micelles. It is related to the position and surroundings of certain molecules in the micelle; some of them are not entirely frozen since they are solubilized into the TDC micelles. This can be seen from the zoomed snapshots of the separate micelles shown in Figure S9 of the Supplementary Materials. Naturally, being four carbon atoms shorter, the studied distance in C14:0 is the shortest, and the standard deviation is 8 ± 1% of the average value.

In addition to the strong hydrophobic interactions acting between the C18:0 chains, the temperature is also an important parameter affecting the attraction between the molecules. The temperature of 37 °C is much lower than the melting point (50 °C) [7,24] of C18:0, and that is why we observe its freezing as a crystal. This phenomenon is registered in the experiments of Katev et al., too, where very good correlation between the melting point and the solubilization capacity of the mixed micelles with various chain lengths of the lipids is demonstrated [7]. In this case, the aqueous media is highly inappropriate for these molecules, and that is why they are solubilized into TDC micelles as nanocrystals in the simulations or they precipitate at the bottom of the container in the experiments. Consequently, under these conditions, they do not take part in the solubilization of the drug molecules. Even more, the competitive solubilization of C18:0 and the hydrophobic drugs into the BS micelles might take place. The situation is slightly different for C14:0. Its interaction with TDC is the same as that of C18:0, but the FA-FA interaction is much weaker than among C18:0 chains, which is related to the hydrophobic attractions on the one hand and to the melting point on the other hand, as mentioned above. The melting of sodium myristate takes place at 25 °C [7,24], which is lower than the working temperature, indicating its fluid state during the simulations and in the experiments. This fact may be outlined as a reason for the positive effect of C14:0 on the solubilization of the hydrophobic drugs. Being more flexible as a fluid than long-chain saturated FAs and having a shorter chain, myristate is able to host more hydrophobic molecules into the mixed micelles. The interactions between TDC molecules are also the same as in the presence of C18:0.

From these comparisons of the intermolecular interactions of the two saturated FAs with different chain length, we can conclude that the main factor affecting the solubilization capacity of the mixed micelles of FAs and BSs is the strength of the interactions between the lipid chains, which form the hydrophobic core of the micelles. When these interactions are comparable to the interactions between TDC molecules, they tolerate penetration of new hydrophobic guests into the micelle core. Unlike that, when there is very strong attraction between the lipid chains, these micelles are not able to solubilize new molecules. That is why the solubilization ratio of the system with C18:0 is almost the same as that of the system without excipient [7]. Along these lines, the effect is much more pronounced for the unsaturated chains because the intermolecular interactions between them are much weaker than between the saturated chains, and their melting point is significantly lower than 37 °C (<20 °C).

It is known in the literature [1,9] and from our previous work [10] that in addition to the hydrophobic forces, there are hydrogen bonds between BS molecules stabilizing the obtained aggregates. That is why we analyzed them and those between TDC and FAs in the current study, too (Figure S11 in the Supplementary Materials). It is found that at least one hydrogen bond is formed between TDC with itself and with FAs. There is no difference between the various FAs, neither with respect to the population of the HB nor to their number. The reason for these similarities between the lipids is related to the fact the HBs are formed between the COO groups of FAs and the taurine tails of TDC, which are located at the same position in the micelles (pointing toward water). However, these HBs are not very strong because their length in terms of donor–acceptor distance is about 0.28 nm (Figure S11B,D in the Supplementary Materials). Therefore, the HBs facilitate the formation of mixed micelles between TDC and FAs, but the interaction between the tails determine the ability of these mixed micelles to solubilize drug molecules therein.

## 3. Molecular Models and Computational Protocol

**Figure 9:**
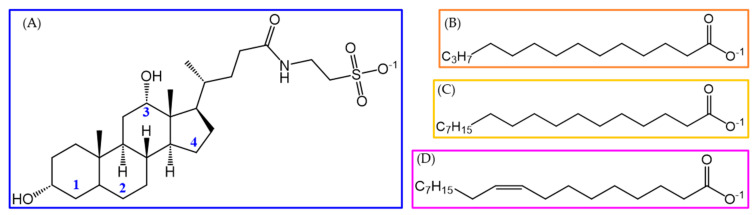
The chemical structures of the studied molecules: (**A**) taurodeoxycholate, TDC, with notation of the cyclic fragments; (**B**) myristate, C14:0; (**C**) stearate, C18:0; and (**D**) oleate, C18:1.

Four systems were built and simulated with fully atomistic MD in explicit aqueous medium, containing (1) taurodeoxycholate, TDC; (2) TDC + sodium myristate, C14:0; (3) TDC + sodium stearate, C18:0; and (4) TDC + sodium oleate, C18:1. The chemical structures of the used molecules are presented in Figure 9. The model systems for the simulations were constructed by mimicking the experimental conditions in in vivo experiments [7]. The concentrations of the main components (TC and FAs) were increased 5 times (from 10 mM to 50 mM), but the ratio between them (1:1) and the electrolyte concentrations (137 mM Na^+^ and 10 mM K^+^) were kept identical to the experimental ones. The higher concentrations were used in order to have sufficient number of molecules for the formation of more than one micelle for better statistical analysis on the one hand and to have less water molecules in order to render the models computationally feasible (ca. 325 × 10^3^ atoms) on the other. Note that these concentrations are significantly above the CMC of both TDC and FAs. The experimentally determined CMC for TDC is about 2 mM according to different literature sources [5,16,25], it is 2 mM for C18:1 [7], and it is estimated in the range 0.14–0.33 mM for C14:0 [7]. Keeping in mind that the CMC of saturated FAs decreases 2–3 fold for each CH_2_ group added to the acyl chain [23], C18:0 was expected to have significantly lower CMC, too. These concentrations of the ingredients corresponded to 100 TDC, 100 FA anions neutralized with 200 Na^+^, and the electrolytes 278 NaCl and 20 KCl added as ions in the aqueous solutions with ca. 103,500 water molecules in a periodic box with edge size of 15 nm along each axis. The BS, FA molecules, and electrolytes were described with the force field AMBER99 [26], and the model TIP3P [27] was used for water. The force field parameters for TDC were derived in our previous work [10], and the already existing optimized geometry file was directly used for the construction of the TDC model systems herein. The molecules of the FAs were subjected to the following procedure: (i) conformational search with AMBER99 in water and geometry optimization of the representative structures with the DFT functional B3LYP [28,29] and basis set 6-31G* [30] in vacuo; (ii) calculation of the atomic partial charges via applying the RESP (Restrained ElectroStatic Potential) procedure [31,32], where the charges were fit to the quantum mechanical electrostatic potential of each molecule, generated at the HF/6-31G* level and the final RESP charges were averaged over the most stable conformers of each molecule; (iii) import of the molecules into the force field library, as the force field AMBER99 contains all necessary parameters for these molecules.

Initially, a model system containing TDC molecules only, randomly placed in the simulation box as monomers (Figure S12A in the Supplementary Materials), was simulated to monitor their aggregation and to obtain micelles, which were used for further construction of the mixed systems with FAs. The obtained TDC micelles after 200 ns of production simulation were taken, and FA monomers were randomly added to them in the same simulation box (Figure S12B in the Supplementary Materials). Having the desired surfactant molecules in all model systems, water molecules and electrolytes ions were further added (Figure S12A in the Supplementary Materials). The following computational protocol was employed for all systems: (i) energy minimization; (ii) heating to 310 K; (iii) relaxation for 1 ns at 310 K maintained with Berendsen thermostat [33] with coupling time of 0.1 ps; and (iv) production runs for 300 ns with time step 2 fs in NVT ensemble. The algorithm leapfrog was used to integrate the equations of motion. The lengths of all hydrogen-containing bonds were fixed with LINCS [34] (for the TDC and FAs) and SETTLE [35] (for the water molecules). The non-bonded interactions were described by Lennard–Jones potential and Coulomb term, respectively, at cutoff 12 Å with a switch function initiated at 10 Å. Long-range electrostatic interactions were evaluated with PME [36,37]. Snapshots were saved in the trajectories at intervals of 10 ps. At least two independent simulations were run with different initial configuration of the molecules in the periodic box for reproducibility of the obtained results with respect to the aggregation kinetics and aggregation number. Equilibration of the systems was verified by monitoring the evolution of the total energy and temperature of the systems and RMSD of the BS and FA molecules. All these parameters fluctuated about constant average values during the production runs, which confirmed that thermodynamic equilibrium was attained. However, the detailed analysis for characterization of the obtained micelles was applied only for the last 10 ns of the production runs, where constant value of the aggregation number was reached, and on the entire trajectory to monitor the aggregation kinetics. The program package GROMACS 2021.3 [38] was used for all simulations and analysis and VMD for visualization of the trajectories [39].

The kinetics of aggregation was determined by cluster analysis, carried out with the method of single linkage, which used similarity of atomic coordinates to assign molecules to an aggregate at cutoff 0.28 nm. The latter has the meaning of the largest interatomic distance between the closest atoms of neighboring molecules in a cluster. This value was determined in our previous study [10] from radial distribution functions (RDFs) between the centers of mass of neighboring BS molecules within stable dimers of bile salts. To characterize the obtained stable micelles in terms of composition, ordering of the molecules, size, and shape, separate sub-trajectories containing single micelles were extracted. Analysis of the RDF, radius of gyration, and moments of inertia was performed for each micelle. An approach using the cumulative RDF was used for detailed determination of the composition of the analyzed aggregates. To evaluate the contribution of the hydrophobic moieties in the micelles with different aggregation numbers, the solvent-accessible surface areas (SASAs) were calculated. The obtained results are presented in the text as average values with standard deviations calculated for all micelles in a given system. In case of mixed systems of TDC and FAs, only the micelles containing both types of molecules were taken into account for the calculation of aggregation umber and detailed analysis. To determine the interactions between each kind of molecules forming a micelle, the RDF and hydrogen bonds were calculated. More details about these analyses are given in Section 2, where the respective results are presented.

## 4. Conclusions

Fully atomistic MD simulations of model systems containing the bile salt taurodeoxycholate and three different fatty acids (C14:0, C18:0, and C18:1) are simulated to monitor their aggregation behavior. An analysis of the morphology of the obtained micelles and of the interactions between the molecules therein is performed.

It is found that TDC forms secondary micelles with an aggregation number of 19 ± 2 and a radius of 1.25 ± 0.07 nm through the coalescence of primary micelles driven by the need to reduce the SASA of the hydrophobic part. The mechanism of the micelle formation is revealed and illustrated in detail. In the presence of fatty acids, mixed micelles are formed with an average aggregation number of 25, independently of the used FAs. The radius of gyration of the mixed micelles increases from 1.35 ± 0.20 nm for TDC + C14:0 to 1.39 ± 0.17 nm for TDC + C18:0 and to 1.46 ± 0.14 nm for TDC + C18:1. The chains of the saturated FAs are solubilized inside the TDC micelles, whereas the double bond of C18:1 interacts with the hydrophobic part of TDC and facilitates the formation of mixed micelles. Moreover, there is significant interaction between the C18:0 molecules, which crystallize inside the micelles due to their melting point being higher than 37 °C. The interaction between the C14:0 molecules is weaker than that between the C18:0 ones, but some ordering is observed. It means that the solubilization of new molecules into these micelles will consume significant energy. However, there are no ordering and strong interactions between C18:1 molecules involved in a mixed micelle with TDC; its RDF profile is typical of fluids. There are hydrogen bonds formed between both pairs of molecules TDC-TDC and TDC-FA, but they are almost the same for all systems. 

The main novelties of this work are the following: (1) The first is the much longer characteristic time for secondary micelle formation than that for primary micelle formation due to different mechanisms: the incorporation of monomers into existing aggregates for primary micelles and the merging of primary micelles for secondary micelles. The latter part of the mechanism is reported for the first time. (2) The second is the detailed structural information on mixed micelles formed from bile acids and fatty acids; the presence of a double bond in oleic acid facilitates its integration with the bile acid, leading to the formation of a fluid layer within the micelle core, whereas stearic acid forms nanocrystals in the interior of the micelles. It is also highlighted that TDC and oleic acid form homogeneous mixed micelles, unlike glycocholate and oleic acid [14]. This indicates the importance of the particular representatives of the two classes of compounds needed to achieve a ‘perfect’ match at the molecular level. The differences in the distribution of the molecules in the mixed micelles and in the intermolecular interactions between the FA and the bile salt may explain the higher solubilization capacity of the mixed micelles with C18:1 than with the saturated ones [7]. Its flexibility and fluidity create a very welcoming interior for the hydrophobic guest molecules.

## Figures and Tables

**Table 2:** Partial RESP charges of the first 6-membered cycle of TDC and C9–C10 methylene groups in C14:0 and C18:0 and methine groups in C18:1, their product, and the product ratio between FAs and C18:1.

Molecule		Partial RESP Charge	*q*_1_ *q*_2_	*q*_1_*q*_2_(C18:1)/*q*_1_*q*_2_(FA)
TDC	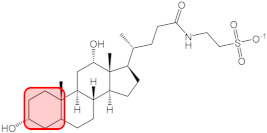	*q*_1_ = 0.492	0.242	-
C14:0	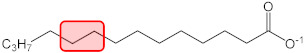	*q*_2_ = −0.053	−0.026	5
C18:0	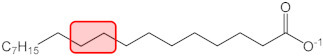	*q*_2_ = −0.007	−0.003	38
C18:1	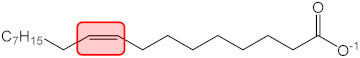	*q*_2_ = −0.246	−0.121	1

## Data Availability

Data are contained within the article and Supplementary Materials.

## Supplementary Materials

The following supporting information can be downloaded at https://www.mdpi.com/article/10.3390/molecules29245897/s1, Figure S1. Configurations of the system containing TDC. Figure S2. Number of monomers and tetramers as of function of time. Figure S3. The distribution of the minimum distances between the TDC molecules. Figure S4. Configurations after 300 ns of the systems containing TDC and FAs. Table S1. The ratio between TDC and FAs and the aggregation number of the formed micelles. Figure S5. The average aggregation number as a function of time in the mixed systems. Figure S6. The cumulative number RDF in %, calculated with respect to the geometric center of each micelle for different residues of the molecules. Figure S7. The RDF between the center of geometry of each molecule type. Figure S8. The RDF between the pair of molecules TDC-TDC and TDC-FA and the maximum value of the RDF divided by the distance at the maximum. Figure S9. Separate snapshots of the mixed TDC and C18:0 micelles at 300 ns. Figure S10. The average distance between C_3_ and C_n-3_ atoms in the hydrocarbon chains of the fatty acids. Figure S11. The population of the number of hydrogen bonds and distributions of the donor–acceptor distances between TDC-TDC and TDC-FA in each micelle. Figure S12. Periodic boxes, which contain randomly placed molecules of TDC and mixed TDC micelles and FA monomers.
