# Supplementary material for: Taurodeoxycholate Aggregation Explored by Molecular Dynamics: Primary-To-Secondary Micelle Transition and Formation of Mixed Micelles with Fatty Acids
Source: Molecules. 2024 Dec 13;29(24):5897. doi: 10.3390/molecules29245897 (PMC11677267; doi:10.3390/molecules29245897)
Supplement: Supplementary file 1 [file molecules-29-05897-s001.zip › molecules-3340019-supplementary.pdf]

Taurodeoxycholate Aggregation Explored by Molecular Dynamics: Primary-to-Secondary Micelle Transition and Formation of Mixed Micelles with Fatty Acids

Fatmegyul Mustan [1,*], Anela Ivanova [2] and Slavka Tcholakova [1,*]

[1] Department of Chemical and Pharmaceutical Engineering, Faculty of Chemistry and Pharmacy, University of Sofia, 1164 Sofia, Bulgaria
[2] Department of Physical Chemistry, Faculty of Chemistry and Pharmacy, University of Sofia, 1 James Bourchier Ave., 1164 Sofia, Bulgaria
[*] Correspondence: fm@lcpe.uni-sofia.bg (F.M.); sc@lcpe.uni-sofia.bg (S.T.); Tel.: +359-28161621 (F.M.)

Supporting information

Figure S1. Configurations of the system containing TDC.
Figure S2. Number of monomers and tetramers as of function of time.
Figure S3. Distribution of the minimum distances between the TDC molecules.
Figure S4. Configurations after 300 ns of the systems containing TDC and FAs.
Table S1. Ratio between TDC and FA and the aggregation number of the formed micelles.
Figure S5. Average aggregation number as a function of time in the mixed systems.
Figure S6. Cumulative number RDF in % calculated with respect to the geometric center of each micelle for different residues of the molecules.
Figure S7. RDF between the center of geometry of each molecule type.
Figure S8. RDF between the pair of molecules TDC-TDC and TDC-FA and the maximum value of the RDF divided by the distance at the maximum.
Figure S9. Separate snapshots of the mixed TDC and C18:0 micelles at 300 ns.
Figure S10. Average distance between C_3 and C_{n-3} atoms in the hydrocarbon chains of the fatty acids.
Figure S11. Population of the number of hydrogen bonds and distributions of the donor-acceptor distances between TDC-TDC and TDC-FA in each micelle.
Figure S12. Periodic boxes, which contain randomly placed molecules of TDC and mixed TDC micelles and FA monomers.

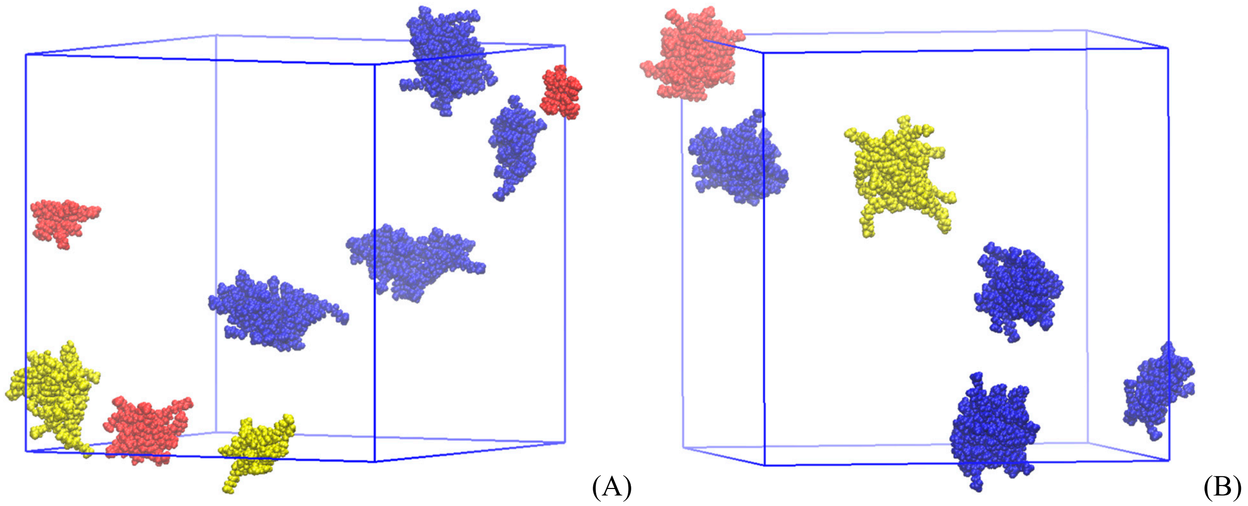

Figure S1. Configurations at (A) 37 ns and (B) 300 ns of the system containing TDC. The red primary micelles at 37 ns correspond to the secondary micelle (red) at 300 ns formed from the same molecules. The same is valid for the yellow micelles. The blue micelles are formed in the early stage of the simulation and remain stable during the entire simulation.

Figure S1 shows that two or three primary micelles with different aggregation numbers at 37 ns are merged into larger secondary micelles at 300 ns. The micelles with 3, 5, and 10 molecules rearrange in the bigger one with 18 molecules (red color) and the micelles with 6 and 14 molecules coalesce to form the one with 20 molecules (yellow color).

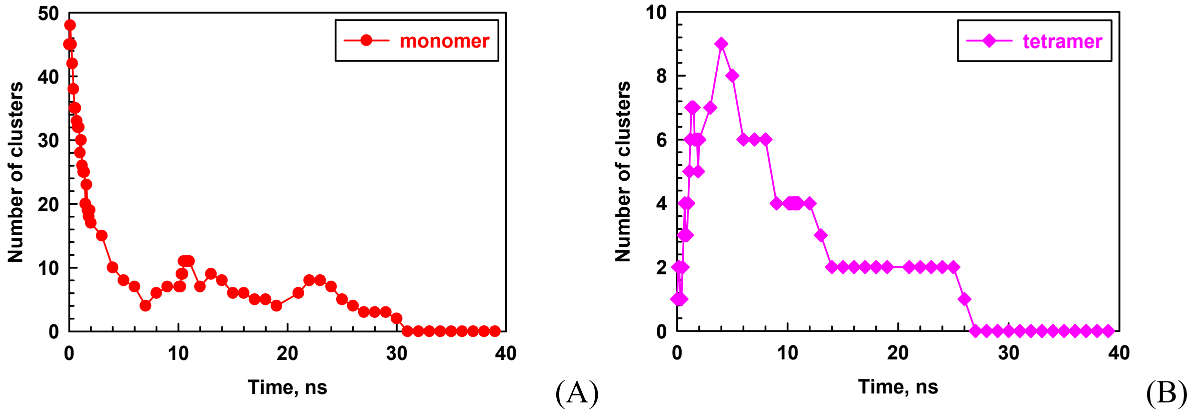

Figure S2. Number of (A) monomers and (B) tetramers as of function of time in the first 40 ns of the simulations of the pure TDC system.

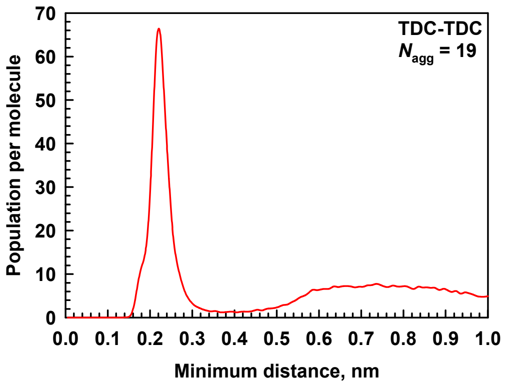

Figure S3. Distribution of the minimum distances between the TDC molecules in the largest micelle with 19 molecules in the period 37-47 ns.

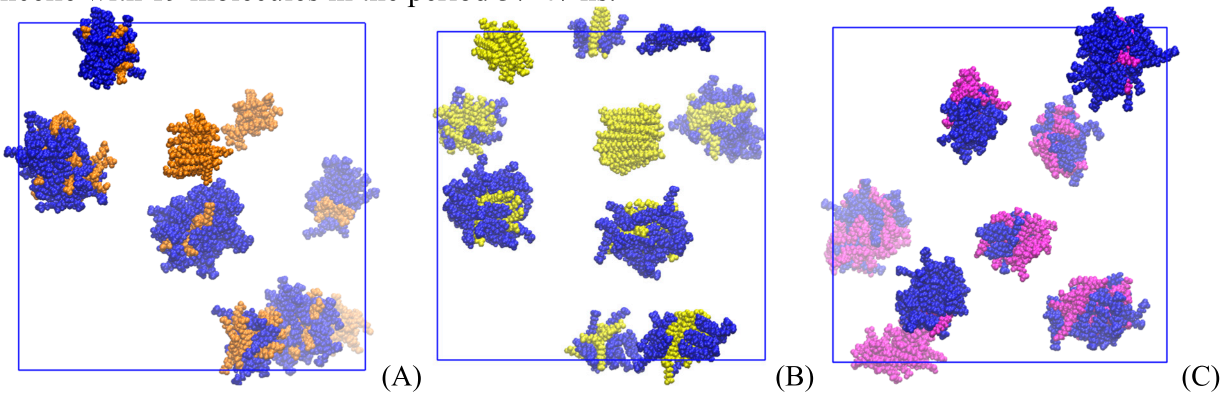

Figure S4. Configurations after 300 ns of the systems containing (A) TDC + C14:0 – orange, (B) TDC + C18:0 – yellow, and (C) TDC + C18:1 – pink.

Table S1. Ratio between TDC and FA and the aggregation number of the formed micelles.

Cluster	FA/(TDC+FA), %			N_{agg}		
	TDC+C14:0	TDC+C18:0	TDC+C18:1	TDC+C14:0	TDC+C18:0	TDC+C18:1
1	34	23	70	50	35	37
2	63	33	13	30	33	31
3	36	48	43	28	31	28
4	24	38	42	21	29	24
5	62	100	100	21	23	22
6	30	39	60	20	18	20
7	100	67	10	15	18	20
8	100	100	67	13	13	18
9	100*			1*		
10	100*			1*		

* Monomers are not included in average N_{agg}

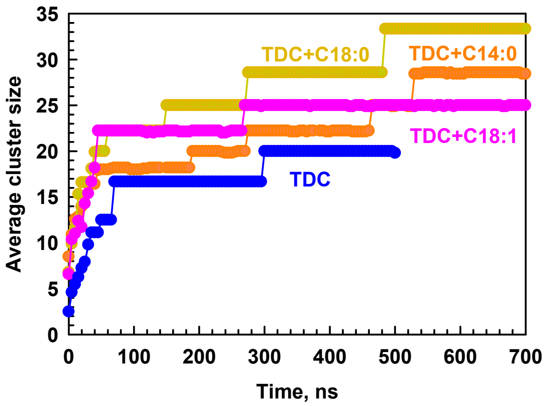

Figure S5. Average aggregation number as a function of time in the systems of TDC only (blue circles) and in the presence of fatty acids: C14:1 (orange circles), C18:0 (yellow circles), and C18:1 (pink circles) for a single MD simulation.

Increase of the simulation time to 700 ns led to further increase in the average cluster size in the systems with saturated fatty acids up to 29 and 33 for C14:0 and C18:0, respectively, whereas it remained constant at 25 for C18:1 for more than 400 ns. Nevertheless, these values are close to the range of the deviation of the sizes obtained up to 300 ns (see **Table 1** of the main manuscript).

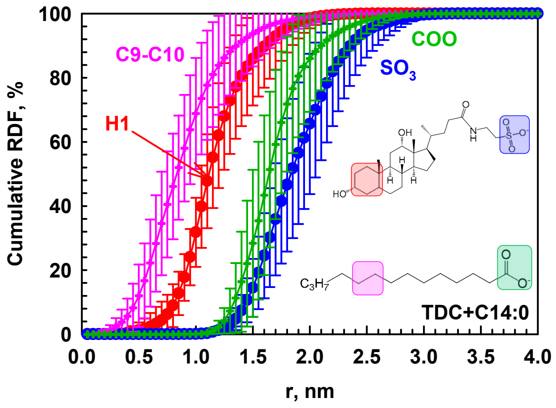

(A)

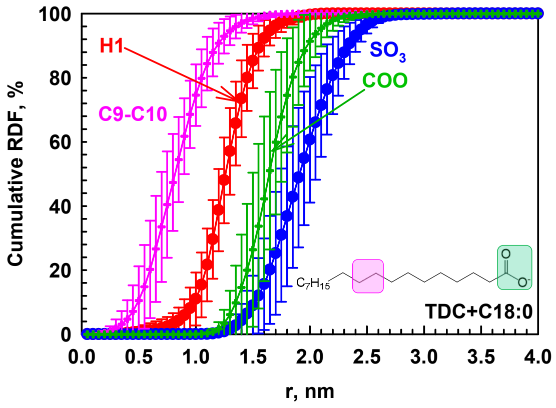

(B)

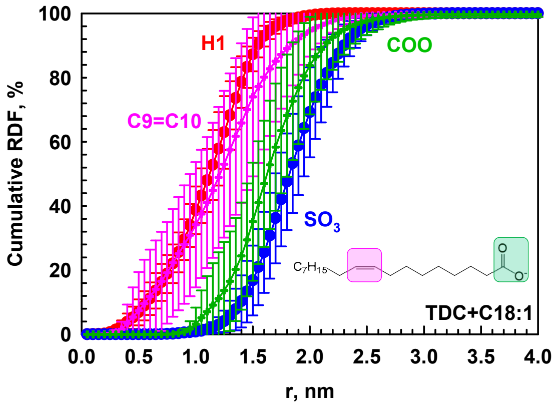

(C)

Figure S6. Cumulative number RDF in % calculated with respect to the geometric center of each micelle for SO_3 – blue circles and first 6-member ring (*H1*) – red circles in TDC, and for COO – green crosses and C9-C10 – pink crosses in FA in the systems of (A) TDC+C14:0, (B) TDC+C18:0, and (D) TDC+C18:1.

C9-C10 are the atoms bound by a double bond in C18:1 and the corresponding ones at the same distance from COO in C14:0 and C18:0 molecules.

The most probable positions relative to the center of the micelle for COO^- and SO_3^- are very similar to each other for all mixed micelles: 1.50±0.08 nm and 1.70±0.16 nm for TDC+C14:0; 1.60±0.12 nm and 1.85±0.09 nm for TDC+C18:0; 1.52±0.22 nm and 1.71±0.16 nm for TDC+C18:1 and very close to the most probable position for SO_3 group in TDC micelles which is 1.55±0.16 nm.

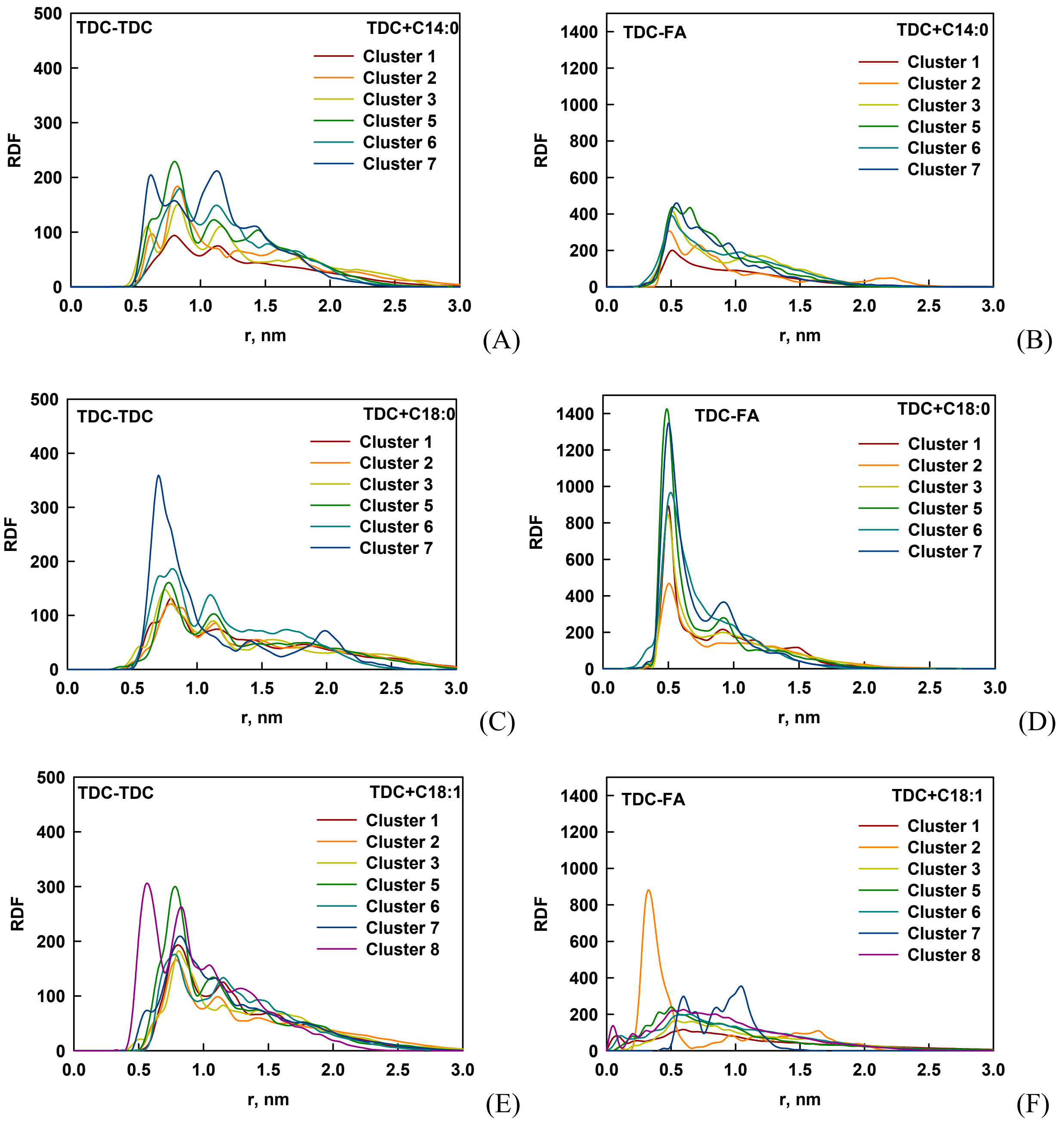

Figure S7. RDF between the center of geometry of each molecule type: (A, C, E) TDC-TDC and (B, D, F) TDC-FA in each micelle in the mixed systems of (A, B) C14:0, (C, D) C18:0, and (E, F) C18:1.

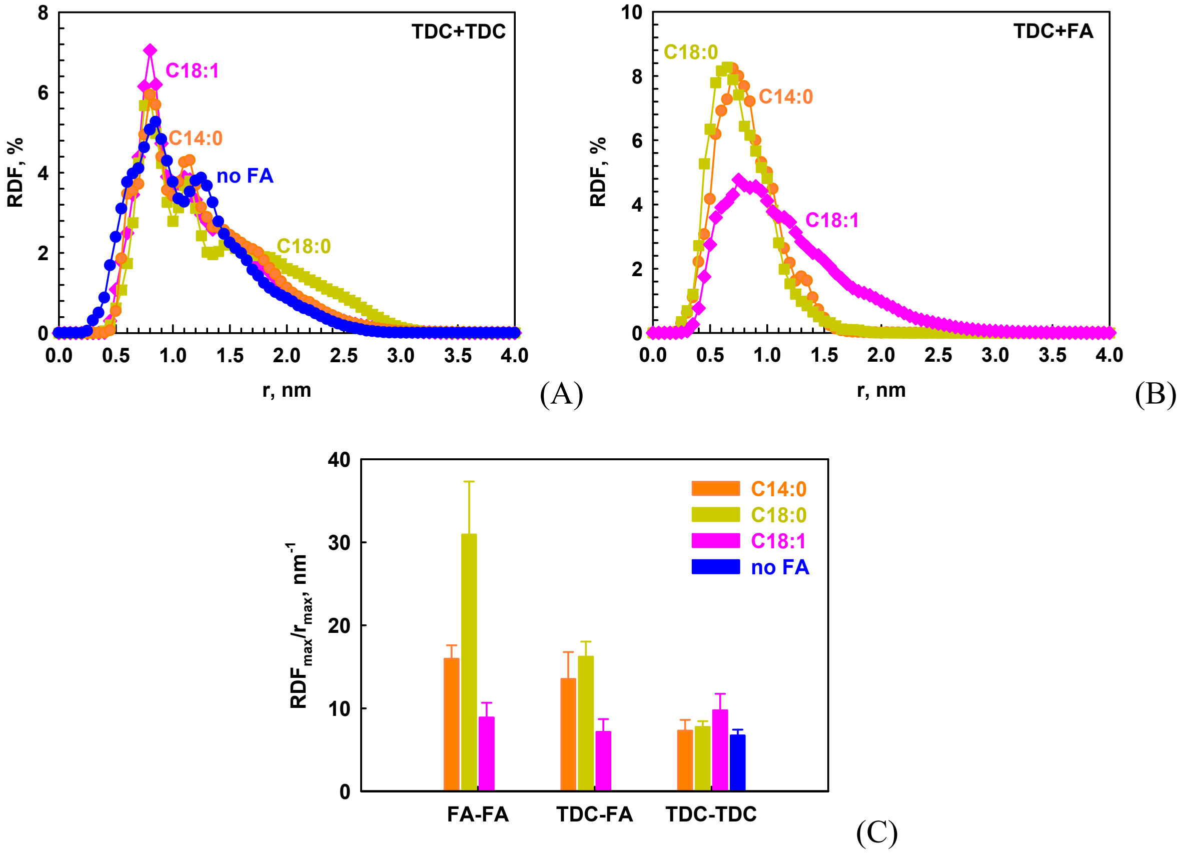

Figure S8. RDF between the center of geometry of (A) TDC-TDC and (B) TDC-FA molecules in the systems without FA (blue circles) and with C14:0 (orange circles), C18:0 (yellow squares), C18:1 (pink rhombi). (C) Maximum value of the RDF divided by the distance at the maximum RDF between FA-FA, TDC-FA, and TDC-TDC calculated for each micelle separately and averaged.

The interactions between TDC molecules are similar in the various model systems (**Figure S8A** of the main manuscript). It is found that there is no significant change in the interaction between TDC molecules upon addition of FA, the profiles are very similar, positioned at the same distance of 0.9 nm. This means that incorporation of the FA does not affect significantly the hydrophobic interactions between TDC molecules, independently on the changes in their morphology, especially in the presence of saturated FA. For better illustration of the differences in the strength of the interactions between the molecules, the maximum of the RDF (RDF_{max}) divided by the respective distance (r_{max}) is plotted as bar chart in **Figure S8C**. It is evident that in the presence of saturated chains, their interaction with TDC and those between TDC and TDC are practically the same, but the FA-FA attraction is much stronger for C18:0.

The width of the RDF peaks is the smallest for C18:0, w=0.16±0.02 nm and the largest for C18:1, w=0.90±0.21. The much larger standard deviation in case of unsaturated tails is one more indication for their much higher flexibility and fluidity than the saturated tails. Moreover, to

determine that flexibility quantitatively, we calculated the distances between the third carbon atoms from both sides of the hydrocarbon chains of the lipids for all molecules separately (**Figure S10** in the SI). It turned out that there is a big difference of 0.33 nm between these distances in the saturated and unsaturated chain, 1.41±0.07 nm vs. 1.07±0.05 nm averaged for all molecules. In addition, the standard deviations of the values for all unsaturated molecules are 17±4 % from the average values, whereas it is only 6±3 % for the saturated chain.

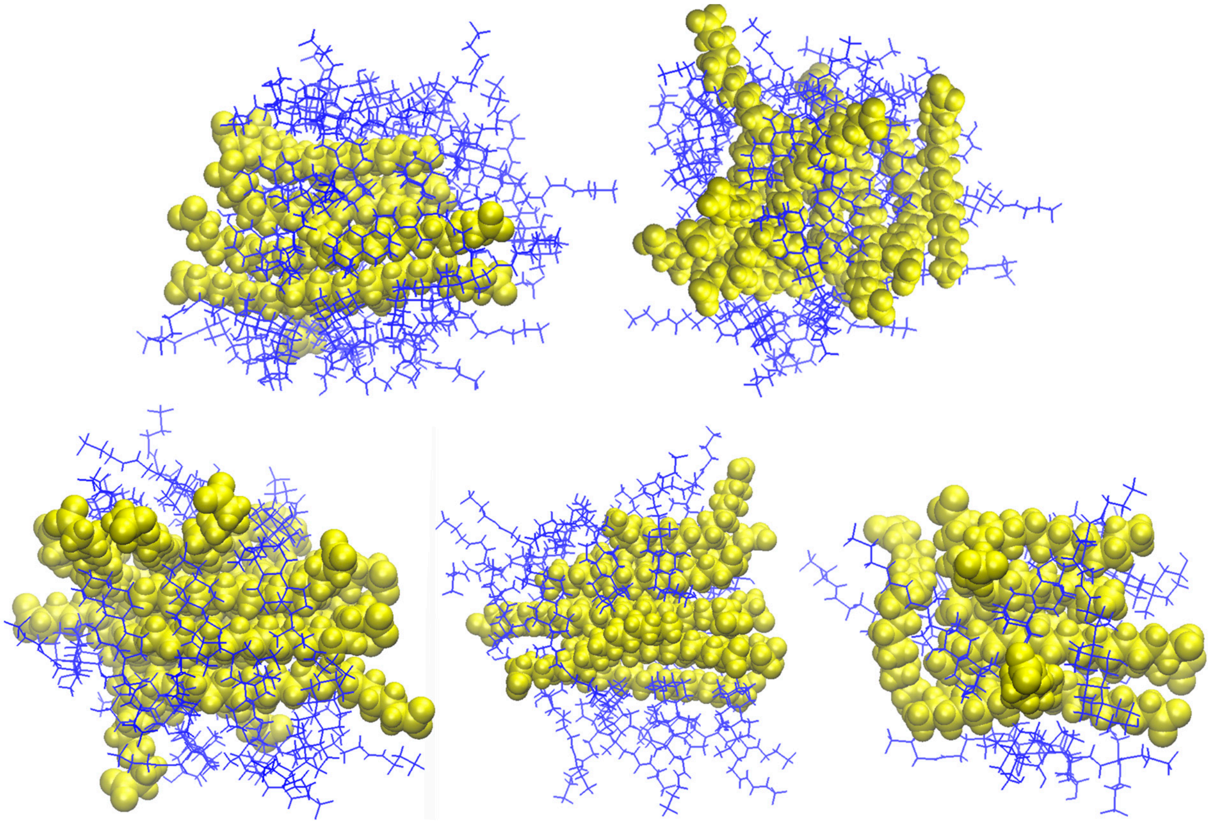

Figure S9. Separate snapshots of the mixed TDC (blue lines) and C18:0 (yellow balls) micelles at 300 ns.

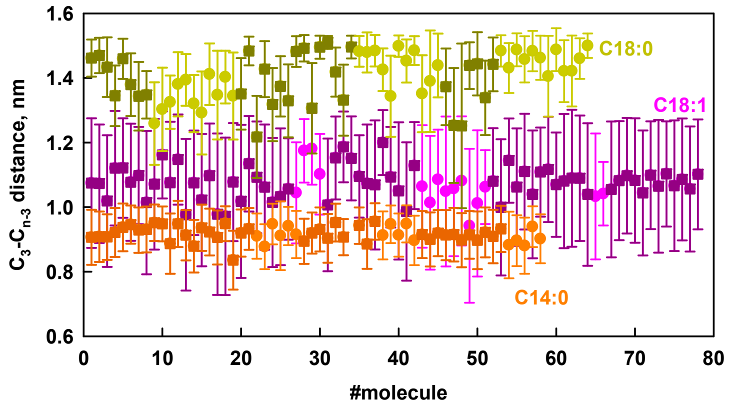

Figure S10. Average distance between C_3 and C_{n-3} atoms in the hydrocarbon chains of the fatty acids: C14:0 – orange symbols, C18:0 – yellow symbols, and C18:1 – pink symbols calculated in the last 10 ns of the simulation for the molecules in the mixed micelles with TDC. The altering colors and symbols for each FA correspond to different micelles: 1, 3, 5, 7 – dark squares and 2, 4, 6 – light circles.

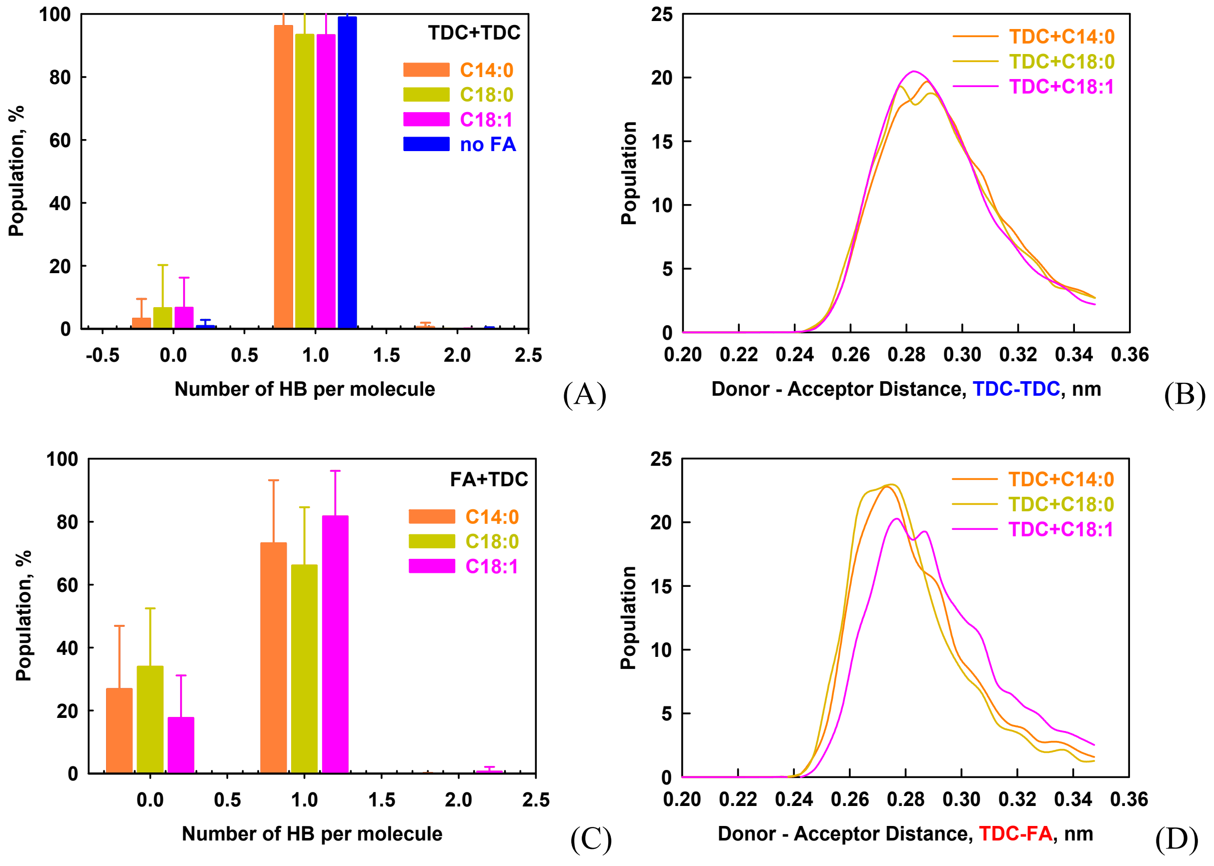

Figure S11. (A,C) Population of the number of hydrogen bonds and (B,D) distributions of the donor-acceptor distances between (A,B) TDC-TDC and (C,D) TDC-FA in each micelle formed in the TDC systems with C14:0 (orange), C18:0 (yellow), C18:1 (pink), and without FA (blue). The number of HB is scaled to the number of the given molecules in each micelle.

Because of the different number of molecules in the micelles, the calculated number of HB in a given micelle is normalized per single molecule to compare all systems accurately (**Figure S11**).

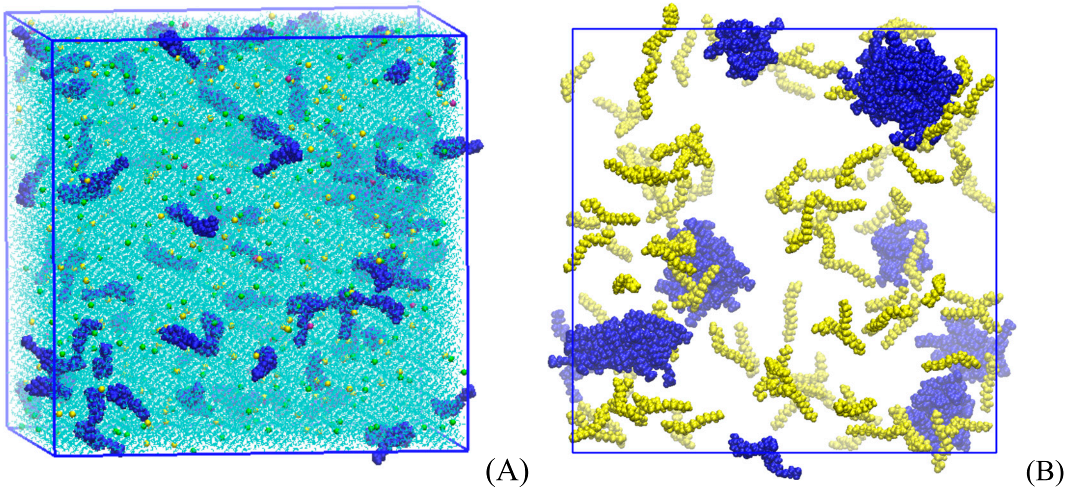

Figure S12. Periodic boxes, which contain randomly placed molecules of (A) TDC – blue and (B) mixed TDC micelles and FA monomers (yellow). In the pure TDC system (A) the ions and water are also visualized: electrolytes ions: Cl^- – yellow, Na^+ – green, K^+ – purple and water – cyan.
